# Syria’s Silent Emergency: Childhood Malnutrition as a Threat to National Recovery

**DOI:** 10.7759/cureus.100335

**Published:** 2025-12-29

**Authors:** M Ihsan Kaadan, Basel Tarab

**Affiliations:** 1 Division of Cardiology, Department of Medicine, Rush University Medical Center, Chicago, USA; 2 Center for Bioethics, Harvard Medical School, Boston, USA; 3 Woods College of Advancing Studies, Boston College, Boston, USA; 4 Patient Experience, Winchester Hospital - Beth Israel Lahey Health, Winchester, USA

**Keywords:** anemia and stunting, fortification, malnutrition, national recovery, syria

## Abstract

Childhood malnutrition in Syria has become an underrecognized threat to the country’s recovery. Recent data indicate high rates of anemia and stunting among children under five years, especially in rural areas, reversing global progress and undermining both physical and cognitive development. These deficits carry long-term economic consequences, including reduced productivity and persistent intergenerational poverty. The crisis is preventable. Syria once maintained a national food-fortification program, and similar efforts in neighboring countries have demonstrated effectiveness even under economic strain. Reintroducing fortified bread, still the country’s main staple, would offer a low-cost, high-impact intervention capable of improving child health on a large scale and restoring public confidence. Addressing malnutrition is therefore not only a public-health priority but also a critical step toward national recovery and the future stability of Syria’s next generation.

## Editorial

In conversations about Syria’s recovery, both in the academic and industrial spheres, much is said about the need to rebuild hospitals, restore infrastructure, and re-establish supply chains. Yet what remains largely unspoken is the quiet crisis affecting Syria’s youngest generation. A 2025 cross-sectional national survey conducted in three public health centers in Syria reported that one in four children aged one to five years is affected by anemia, and one in three experiences growth impairment [1]. Factors contributing to these numbers include poverty, limited maternal education, and geographically uneven food availability [1]. This crisis threatens to define the country’s future as profoundly as any physical destruction, if not more.

WHO estimates from 2019 show that nearly one-third of Syrian children under five suffer from anemia, with rates rising to more than 75% in rural areas [2,3]. These figures are among the highest in the region, exceeding those of Iraq and Lebanon over the past decade, despite their own protracted crises [2]. Globally, anemia has decreased in the past decade, but the trend in Syria has moved in the opposite direction. The regional WHO office reports that almost one in three Syrian children under five is affected by stunting [4]. This is not just a nutritional problem; it is a developmental one. Stunting and anemia weaken immunity, increase vulnerability to infections, and adversely affect cognitive development. The result is a generation less equipped to learn, work, and contribute to rebuilding the nation they will inherit.

The health and human costs are staggering, but the economic toll will be equally severe. Iron-deficiency anemia reduces concentration and learning potential, directly undermining productivity in adulthood. Stunting also has a devastating impact. The World Bank estimates that countries lose, on average, 7% of their GDP per capita because they failed to eliminate stunting among children before they entered the workforce [5]. Addressing the stunting issue for Syrian children will also provide them with an economic advantage in the future at the individual level. Studies have shown that a 1 cm increase in height is associated with a 4% increase in wages for men and a 6% increase for women [6]. For a country already struggling with mass displacement, unemployment, and inflation, such losses could entrench a cycle of poverty that persists long after the conflict ends.

What makes this crisis particularly disheartening is that it is entirely preventable. Fortifying basic foods with iron and folic acid is one of the most effective public health measures available. More than 80 countries have implemented such programs, reducing anemia rates by 2-3% each year [7]. Syria once had a similar program. It began in 2003 but collapsed following the outbreak of war in 2011. Bread, being the main source of caloric intake in Syria, is the most obvious medium for fortification. Today, it is estimated that fewer than 5% of bread loaves are fortified [8]. Meanwhile, countries across the region, including Jordan, Egypt, Oman, and Saudi Arabia, have shown that national fortification can be implemented successfully even under economic constraints. Jordan, for instance, saw anemia rates fall from nearly 29% to 11% in little over a decade [9].

Reviving Syria’s fortification program would not only save lives but also restore a deeper principle: the belief that public health can be rebuilt through deliberate, collective action [10]. Bread remains Syria’s primary staple. Despite years of conflict, mills continue to operate in several provinces, and the infrastructure required for fortification can be reestablished through collaboration with the WHO, UNICEF, and local NGOs. The technical and operational expertise of these international agencies, combined with local knowledge and familiarity with Syria’s specific context, makes this effort achievable. What is required is not innovation, but reconstruction, coordination, and renewed political commitment.

That said, fortification is not a smooth or guaranteed path. A comprehensive regional review of fortification programs in the Middle East has identified persistent challenges, including inadequate coverage and compliance, variable nutrient bioavailability, deterioration of infrastructure, political and policy barriers, and financial constraints [9]. These obstacles are likely to be even greater in Syria, given the duration and intensity of the war. Bread in Syria has long symbolized stability and food security, its presence on every table acting as a tangible indicator of the state’s reliability. Securing this staple is therefore essential not only for improving nutrition but also for restoring public confidence in governance.

In addition to bread fortification, the targeted distribution of energy-dense foods, including ready-to-use therapeutic foods (RUTF), may also be necessary. Evidence from humanitarian settings indicates that such interventions can enhance children’s nutritional status and reduce stunting when combined with population-wide strategies [11].

The moral dimensions of Syria’s nutritional crisis demand explicit attention. Child health is a widely recognized indicator of post-conflict recovery, with implications for human capital, social cohesion, and economic resilience, and cannot be achieved through infrastructure reconstruction alone. Micronutrient fortification offers a low-cost, high-impact intervention that should be implemented early in recovery efforts. Beyond its health benefits, it may also help rebuild public trust by improving the safety and nutritional quality of staple foods. Ensuring adequate nutrition for children is therefore central not only to population health, but also to reestablishing the social foundations necessary for Syria’s long-term recovery.
